# Magnetic Glass Ceramics by Sintering of Borosilicate Glass and Inorganic Waste

**DOI:** 10.3390/ma7085565

**Published:** 2014-07-31

**Authors:** Inès M. M. M. Ponsot, Yiannis Pontikes, Giovanni Baldi, Rama K. Chinnam, Rainer Detsch, Aldo R. Boccaccini, Enrico Bernardo

**Affiliations:** 1 Department of Industrial Engineering, University of Padova, via Marzolo 9, 35131 Padova (PD), Italy; E-Mail: ines.ponsot@dii.unipd.it; 2 Department of Metallurgy and Materials Engineering, Katholieke Universiteit Leuven, Kasteelpark Arenberg 44 bus 2450, B-3001 Heverlee (Leuven), Belgium; E-Mail: pontikes@mtm.kuleuven.be; 3 Colorobbia Research Centre (Ce.Ri.Col.), via Pietramarina 53, 50053 Sovigliana Vinci (FI), Italy; E-Mail: baldig@colorobbia.it; 4 Institute of Biomaterials, Department of Materials Science and Engineering, University of Erlangen-Nuremberg, 91058 Erlangen, Germany; E-Mails: rama.k.s.chinnam@ww.uni-erlangen.de (R.K.C.); rainer.detsch@ww.uni-erlangen.de (R.D.); aldo.boccaccini@ww.uni-erlangen.de (A.R.B.)

**Keywords:** viscous flow sintering, magnetic glass ceramics, induction heating, iron-rich slags, borosilicate glass, cytotoxicity tests

## Abstract

Ceramics and glass ceramics based on industrial waste have been widely recognized as competitive products for building applications; however, there is a great potential for such materials with novel functionalities. In this paper, we discuss the development of magnetic sintered glass ceramics based on two iron-rich slags, coming from non-ferrous metallurgy and recycled borosilicate glass. The substantial viscous flow of the glass led to dense products for rapid treatments at relatively low temperatures (900–1000 °C), whereas glass/slag interactions resulted in the formation of magnetite crystals, providing ferrimagnetism. Such behavior could be exploited for applying the obtained glass ceramics as induction heating plates, according to preliminary tests (showing the rapid heating of selected samples, even above 200 °C). The chemical durability and safety of the obtained glass ceramics were assessed by both leaching tests and cytotoxicity tests.

## 1. Introduction

The recycling of inorganic waste into new usable ceramic products has been a key strategy for environmental protection for the last few decades [1], which has included also efforts to produce glass ceramics from waste [2]. Glass ceramics undoubtedly have constituted an important and established waste-derived product since the 1960s [3]. Nevertheless, it must be acknowledged that the energy-intensive vitrification process at the basis of any glass ceramic manufacturing and the limited applications, mainly as building materials, remain as fundamental issues [2,4].

The direct sintering of mixtures of inorganic waste, including recycled glasses, acting as fluxing agents, is an important alternative to conventional glass ceramics. The products cannot be nominally considered as glass ceramics, since there is no actual vitrification, *i.e.*, melting of an oxide mixture and cooling in an amorphous solid. However, a rich literature supports the classification of such products as “sintered glass ceramics”, owing to the generally observed phase evolution [2,5,6,7]. In fact, recycled glasses, besides promoting the densification by viscous flow sintering, react with the waste, leading to silicate and alumino-silicate crystals similar to those developed by devitrification of waste glasses. The process offers remarkable energy savings, due to the absence of a high temperature (>1350–1400 °C) melting stage and its simplicity.

As widely discussed by Chinnam *et al.* [8], iron-rich waste materials, when incorporated in glass ceramics, demonstrate the potential of turning these waste-derived materials into functional glass-based products, appreciated for their magnetic, electrical and thermal properties. In particular, the magnetic functionality is interesting for the possibility of induction heating, exploited even in non-waste-derived iron-rich glasses and glass ceramics, such as those developed for cancer treatment by hyperthermia [9,10]; in fact, ferrimagnetic particles (such as magnetite crystals, embedded in a glass matrix) may provide intensive heating, owing to energy dissipation upon magnetization cycles [11].

In the present study, we investigated mixtures of pharmaceutical borosilicate glass residues and two iron-rich slags, coming from non-ferrous metallurgy. One of the slags, in particular, contains fayalite, *i.e.*, iron (II) silicate (Fe_2_SiO_4_), a typical crystal phase in many slags from non-ferrous metallurgical processes [12,13]. Whereas fayalite-rich slags have been already applied directly in the elaboration of foamed concrete aggregates [14], they have been used in glass ceramic materials, to the authors’ knowledge, only after a vitrification process aiming at the separation of iron from the glassy matrix [15,16,17].

In this investigation, the mixing of slags with borosilicate glass was successful in yielding dense products by fast sintering at relatively low temperatures (900–1000 °C) with an excellent stabilization of pollutants (e.g., heavy metal ions) present in the slags, as assessed by direct leaching tests and by cytotoxicity tests. These tests using cell culture methods are being proposed to provide reliable data about the safety of waste-derived products, e.g., when they come into direct contact with biological entities. The formation of magnetite, as the main crystal phase in the obtained glass ceramics, was exploited for considering the application of the new glass ceramics in induction heating applications.

## 2. Experimental Section

### 2.1. Materials and Processing Methods

The starting wastes consisted of metallurgical slags labeled S1 and S2. The drive for investigating these particular slags relates to the fact that they are quite typical and can be seen as two end members for non-ferrous slags, *i.e.*, Fe,Si-rich and Fe,Ca,Si-rich. The slags were mixed with recycled borosilicate glass, from the manufacturing of pharmaceutical containers (BS), in the following proportions (expressed in wt%): 75 BS-25 S1, 50 BS-50 S1, 75 BS-25 S2 and 50 BS-50 S2. The chemical composition of the starting wastes (determined by X-ray fluorescence spectrometry, Philips PW2400 XRF, Almelo, The Netherlands), as well as of borosilicate glass (already reported in the literature [18]) is reported in Table 1. The slags were first wet ball milled for 30 min (water in an amount of 50% of the total solid), in order to reach a maximum particle size of about 100 µm, and then borosilicate glass was added, in the form of powders with a maximum particle size of 200 µm. The slag/glass mixtures were homogenized by a secondary ball milling process (400 rpm, for 5 min) and left to oven dry at 110 °C overnight. The dried slips were passed through a sieve, in order to get fine granules of about 200 μm in diameter.

**Table 1:** Chemical composition of starting slags and recycled glass. S, slag; BS, borosilicate glass.

Component	S1	S2	BS
Chemical composition (wt%)			
SiO_2_	29	24	72
FeO	52	32	–
Al_2_O_3_	4	6	7
CaO	2	21	1
MgO	1	1	–
Na_2_O	<1	<1	6
K_2_O	<1	<1	2
ZnO	7	7	–

Sintering experiments were first performed on disc samples, with a diameter of about 30 mm and a height of 2 mm, obtained by the uni-axial pressing at 40 MPa of fine powders in a cylindrical steel die at room temperature, without any binder. The samples were directly inserted in the furnace preheated at 900 °C, 950 °C or 1000 °C, and left for 30 min. Afterwards, the samples were removed from the furnace and rapidly cooled in air. All sintered samples featured a brown-red color. Secondary sintering treatments, at selected temperatures, were applied on larger prismatic samples with dimensions of about 50 mm × 35 mm × 4 mm, obtained by uniaxial pressing at 40 MPa in a rectangular steel die. The samples were heated at a rate of 40 °C/min and cooled in the furnace after the holding stage (in order to keep the holding time at exactly 30 min, the furnace was first rapidly cooled below 550–600 °C, by leaving the furnace door partially open, and then left to cool naturally).

### 2.2. Microstructural Characterization and Mechanical Properties

The apparent density and the water absorption of the sintered glass ceramics were evaluated by means of the Archimedes’ principle (the results are the average of those of 5–6 samples, for each composition and sintering condition). Evaluations of the amount of residual porosity were performed by means of image analysis, performed by using the ImageJ program package on scanning electron microscopy (SEM, JSM-6300 and JSM-6490, JEOL, Tokyo, Japan) micrographs, employed also for the microstructural studies. In selected cases, a microchemical analysis was performed by means of energy dispersive X-ray spectroscopy (EDS, LINK PentaFET 6699, Oxford Instruments, Abingdon, UK).

X-ray diffraction analyses (Bruker D8 Advance, Karlsruhe, Germany) were performed on powdered samples, employing CuKα radiation (0.15418 nm), in the interval 2θ = 15–60°. Phase identification was achieved by means of the Match! program package (Crystal Impact GbR, Bonn, Germany), supported by data from the PDF-2 (Powder Diffraction File) database (ICDD, International Centre for Diffraction Data, Newtown Square, PA, USA).

The bending strength was measured on small beams of about 30 mm × 2 mm × 3 mm, cut from bigger samples. All beams were carefully polished to a 5-μm finish and chamfered at the edges, by using diamond tools. The elastic modulus was measured by non-destructive resonance frequency testing (GrindoSonic Mk5, Leuven, Belgium). Four point bending tests (24 mm outer span, 8 mm inner span) were performed by using an Instron 1121 UTS (Instron, Danvers, MA, USA), with a cross-head speed of 1 mm/min; each data point represents the average of at least 10 individual tests. Selected polished samples were employed for Vickers micro-hardness tests (Officine Galileo, DG 901 micro-indenter, Florence, Italy) at a low load (3 N).

### 2.3. Chemical Durability Tests

The release of heavy metals was evaluated by application of the Toxicity Control Leaching Procedure (or TCLP): fragments from bending strength tests were placed in an extraction solution consisting of distilled water, with a pH value of about 7, prepared according to the European Standard for waste toxicity evaluation (European Standard EN 12457) for a liquid-to-solid ratio of 10 and softly stirred at 25 °C for 24 h. The resulting solutions were filtered through a 0.6-μm filter and analyzed by using inductively-coupled plasma (ICP, SPECTRO analytical Instruments GmbH, Kleve, Germany).

### 2.4. Cytotoxicity Investigation

The biocompatibility of samples was assessed by cell culture tests, applied on small discs (1 mm thickness, 13 mm diameter) prepared as above, but used after gradual polishing, up to a 350-nm finish, by means of diamond tools. Biocompatibility tests were performed according to both direct and indirect methods [19]. A suspension of mouse embryonic fibroblast (MEF) cells was cultured for 24 h in Dulbecco’s Modified Eagle Medium (DMEM) containing 10% fetal bovine serum (FBS) and 1% of penicillin-streptomycin, at 37 °C, in a humidified atmosphere incubator (air, with 5% CO_2_).

In the direct method, cells were seeded on the samples (selected sintered glass ceramics and soda-lime glass, used as a reference), for an incubation period of 24-h. In the indirect method (elution test), the samples were placed in a separate cell culture medium under standard conditions. Fluid extracts obtained after 1, 2 and 3 days were added to a cultured-cell monolayer. In this way, test cells were supplied with a fresh nutrient medium containing extracts derived from the test and control material. Each culture was incubated for 24 h.

For cell viability measurement, the Water Soluble Tetrasodium Test (WST1) (Sigma Aldrich^®^, Gillingham, UK) was used, as recommended by the manufacturer. The qualitative evaluation was performed using a fluorescence microscope (ZEISS Scope A1, Carl Zeiss Microscopy GmbH, Jena, Germany. Blue fluorescent DAPI (40,6-diamidino-2-phenylindole dihydro-chloride, Roche, Basel, Switzerland) and green calcein were used for staining the nucleus and cytoplasm of the cells.

### 2.5. Induction Heating Tests

Selected discs (5 mm mean thickness and 25 mm mean diameter) were finally subjected to induction heating tests. The samples were placed at the center of the copper coils (9 turns and Φ = 50 mm) of a Nova Star 5kW^®^ power supply (Ameritherm Inc., Scottsville, NY, USA) operating at 21 kA/m with a frequency of 168 kHz. The temperature was measured with an IR thermo-camera (FLIR Thermacam^®^ E65, FLIR System, Boston, MA, USA).

## 3. Results and Discussion

### 3.1. Microstructural Evolution

The temperature necessary for an important densification was set at 900 °C, as a minimum firing temperature for glass/slag mixtures. In fact, because of the presence of secondary phases, some residual porosity could not be eliminated, even operating at 850 °C, despite the possibility of achieving nearly full density at only 700 °C for the pure glass, in good agreement with previous experience on the adopted borosilicate glass combined with alumina platelets, under 15 vol% [18].

**Figure 1:**
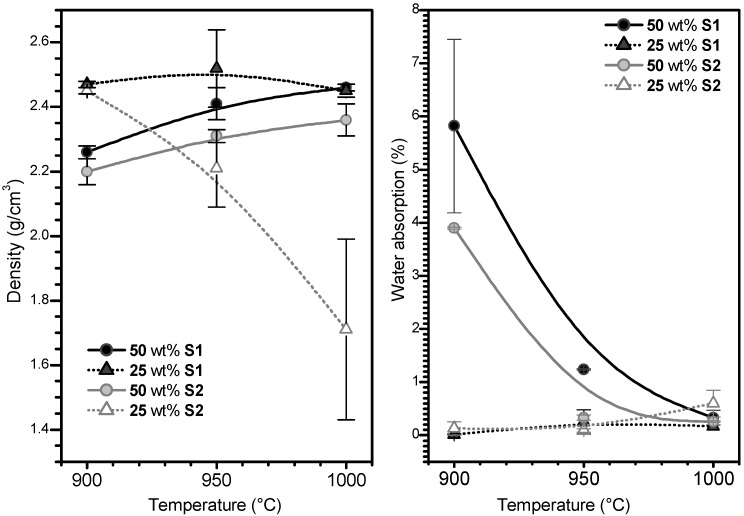
Density and water absorption evolutions with composition and temperature (B-spline lines are provided simply as guides for the readers’ eye).

As shown in Figure 1, both density and water absorption exhibit quite particular evolutions, with increasing firing temperature depending on the composition. The most straightforward trends correspond to the samples containing a significant amount of slag (50 wt% of S1 or 50 wt% of S2), which exhibited a slight increase of density (always below 2.5 g/cm^3^), coupled with a sensible decrease of water absorption. Softened borosilicate glass reasonably “glued” the slag particles, progressively removing the interstitial porosity. Such densification was more efficient with increasing temperature (causing a decrease of viscosity) and, at the outer part of samples, obviously hotter (being closer to the heating elements). In fact, the latter effect is evident on some samples fired at 1000 °C, in which water absorption approached zero and only internal closed porosity remained [20,21].

The samples with low slag content (25 wt% of S1 or 25 wt% of S2) exhibited negligible water absorption, even at 900 °C. The increased glass content evidently caused a more intensive viscous flow with the sealing of external porosity. The density, however, had quite surprising trends; whereas the density of samples featuring S1 slag remained almost stationary, the density of samples containing S2 slag exhibited a remarkable decrease with increasing firing temperature (from 2.45 g/cm^3^, at 900 °C, to 1.7 g/cm^3^, at 1000 °C).

**Figure 2:**
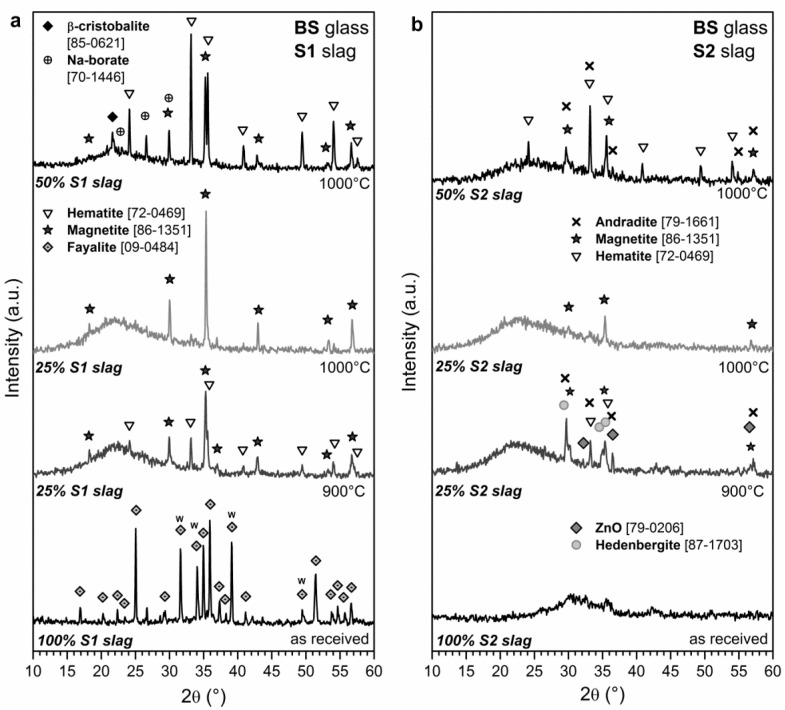
Crystalline phase evolution of glass/slag mixtures based on (**a**) S1 slag; (**b**) S2 slag.

The different behavior of samples with different slags and/or with different slag concentrations is attributed to the specific glass/slag interactions. As demonstrated by the X-ray diffraction patterns in Figure 2, the borosilicate glass did not merely encapsulate the slags in a glass matrix, but promoted phase transformations.

The S1 slag, in the as-received conditions, as shown by Figure 2a, contained fayalite, *i.e.*, Fe(II) silicate (Fe_2_SiO_4_ or 2FeO∙SiO_2_, PDF#09-0484). The intensity of some peaks (labelled with “w”) is actually significantly higher than that of pure fayalite, which could be due to the incorporation of Zn^2+^ ions, with the formation of a solid solution. This is supported by the fact that willemite (Zn_2_SiO_4_ or 2ZnO∙SiO_2_, e.g., PDF#02-1413) effectively possesses strong peaks in the selected positions (label “w” in Figure 2a) and forms solid solutions with fayalite [22,23].

Air, *i.e.*, an oxidative atmosphere, may cause the decomposition (“oxygenolysis”) of fayalite, with the formation of iron oxides, according to the following reactions [24]:

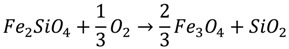
(1)

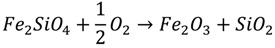
(2)


At 900 °C, with S1 slag present in the amount of 25 wt%, both reactions likely occurred, with the formation of magnetite, *i.e.*, iron oxide with both Fe^2+^ and Fe^3+^ (Fe_3_O_4_, or FeO∙Fe_2_O_3_, PDF#86-1351), and hematite, *i.e.*, iron oxide with only Fe^3+^ ions (Fe_2_O_3_, PDF#72-0469), well visible in the pattern in Figure 2a. At 1000 °C, with the same concentration of S1 slag, magnetite was favored consistently with the high temperature reduction of iron oxides in a viscous mass, associated with oxygen release, as discussed by Appendino *et al.* [25]. It can be noted that crystalline silica is not visible in the patterns for 25 wt% S1 slag: the secondary product of the oxygenolysis reaction was probably dissolved by the borosilicate glass.

Operating with 50 wt% S1 slag, as illustrated by the upper pattern in Figure 2a, magnetite and hematite are confirmed as the main crystal phases. However, in this case, hematite is dominant. This behavior is likely due to more intensive oxygen diffusion, promoting the reaction induced by Equation (2): the reduced viscous flow, associated with the lower glass content, probably did not allow an instantaneous sealing of the slag particles from atmospheric oxygen.

The minor phases detected in the X-ray diffraction pattern of sample with 50 wt% S1 slag are quite interesting. Whereas sodium borate (Na_2_B_6_O_10_ or Na_2_O∙3B_2_O_3_, PDF#70-1446) could be attributed to the crystallization of the distinctive boron-rich phase of borosilicate glasses (borosilicate glasses typically exhibit phase separation [26]), the observed variety of crystalline silica, *i.e.*, β-cristobalite, could be seen as proof of the glass slag interaction. The detected phase could form by incomplete dissolution of silica from oxygenolysis, and it could be stabilized in the high temperature variant (β-phase) by incorporation of alumina, relatively high in the adopted borosilicate glass [19] (SiO_4_ tetrahedra may be partially replaced by AlO_4_ tetrahedra, with extra cations, such as Na^+^, Ca^2+^, Cu^2+^ and Sr^2+^, from both glass and slag, compensating for the charge variation from Si^4+^ to Al^3+^) [27].

As shown by Figure 2b, S2 slag was practically amorphous in the as-received conditions; the mixing with borosilicate glass yielded again iron-rich phases, comprising both Fe^2+^ and Fe^3+^, but mainly in association with calcium oxide. This is not surprising, owing to the much more significant content of CaO in S2 slag compared with S1 slag. With limited slag content (25 wt%), the firing at 900 °C caused the separation of calcium-iron silicates, such as andradite (Ca_3_Fe_2_(SiO_4_)_3_, PDF#79-1661), featuring Fe^3+^ ions, and hedenbergite (CaFeSi_2_O_6_), featuring Fe^2+^ ions. Magnetite and hematite, dominant with S1 slag, are reasonably still present, in the form of traces, as well as zinc oxide (ZnO, PDF#79-0206). As observed for S1 slag, a temperature increase, starting from the same slag concentration, favored the formation of magnetite, with the rest of the crystal phases dissolved by the borosilicate glass. The remarkable reduction of density could be due to the previously mentioned oxygen release, in turn provided by the Fe^3+^/Fe^2+^ reduction, causing some foaming. Indeed, due to the higher content of network modifiers provided by the S2 slag, it is expected that the glass phase would be less viscous and more prone to foaming, than in the case of the S1 slag.

Operating with a high content (50 wt%) of S2 slag, in analogy with the samples made with the S1 slag, the formation of more oxidized phases was favored. In fact, magnetite is present (see the upper pattern of Figure 2b) coupled with andradite and hematite.

### 3.2. Mechanical and Functional Characterization

Table 2 summarizes the properties of samples based on S1 and S2 slag, sintered in the form of rectangular tiles. In this case, in order to mimic industrial firing, heating was performed at a steady rate (40 °C/min), and the samples were not removed from the furnace directly at high temperature, to avoid thermal shock. We focused on the conditions (concentration, temperature) that could maximize the density, on the one hand, and minimize the water absorption, on the other.

**Table 2:** Physical and mechanical properties of selected glass ceramics based on glass/slag mixtures.

Sample	Sintering Temperature (°C)	Apparent density (g/cm^3^)	Porosity (%)	Elastic modulus (GPa)	Bending strength (MPa)	Vickers Hardness (GPa)
25% S1	900	2.34 ± 0.01	7.9 ± 0.5	69.0 ± 14.9	39.6 ± 11.1	5.3 ± 2.1
50% S1	1000	2.39 ± 0.01	16.4 ± 0.5	62.3 ± 13.4	33.0 ± 1.3	5.9 ± 1.1
25% S2	900	2.32 ± 0.01	9.6 ± 0.5	69.3 ± 4.8	37.6 ± 6.2	4.6 ± 0.7
50% S2	1000	2.39 ± 0.01	18.1 ± 0.5	56.8 ± 3.0	32.4 ± 7.6	5.4 ± 1.3

The density values are in good agreement with those referred to small discs, subjected to direct heating. Despite the relatively high residual porosity (inferred from image analysis), varying from 8% to 18%, the obtained glass ceramics compare favorably, in terms of elastic modulus, bending strength and Vickers hardness, with analogous waste-derived glass ceramics [28,29]. Like analogous waste-derived glass ceramics, owing to the negligible water absorption, the developed materials could be used as low-cost building materials.

**Figure 3:**
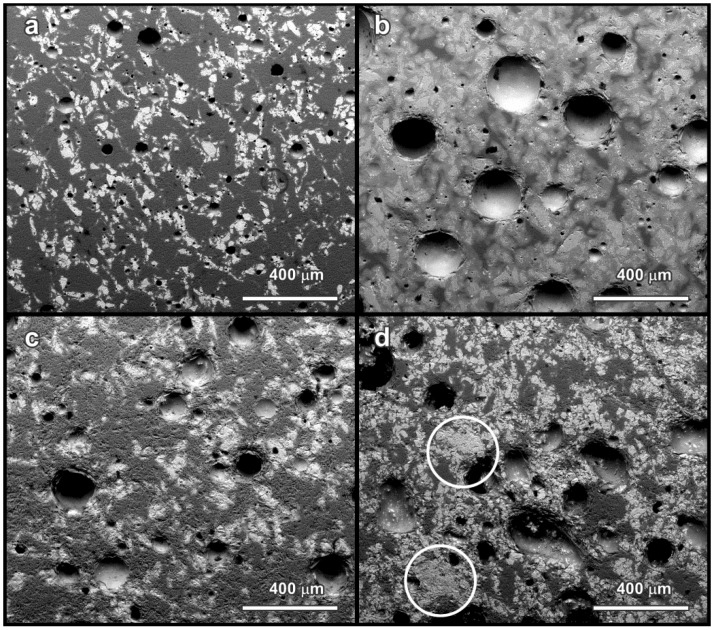
SEM micrographs of selected glass ceramics (polished surfaces of bending bars, cut from rectangular tiles): (**a**) 25 wt% S1, 900 °C; (**b**) 50 wt% S1, 1000 °C; (**c**) 25 wt% S2, 900 °C; (**d**) 50 wt% S2, 1000 °C.

The SEM micrographs in Figure 3 confirm a quite homogeneous distribution of components in the glass ceramic microstructure. In particular, iron-containing phases may be easily distinguished from the light color in the back-scattered electrons images. The homogeneity is particularly important for the stabilization of pollutants. In fact, the chemical homogeneity of a glass ceramic from simple sintering of glass/slag mixture is obviously lower than that of a glass ceramic obtained from melting, solidification of glass and crystallization; pollutants may selectively accumulate in aggregates of slag particles not completely encapsulated in the liquid phase provided by the glass. Except for Figure 3d (see the highlighted areas), showing a sample with 50 wt% S2 slag, sintered at 1000 °C, no large slag-derived aggregates are visible. On the contrary, the sample with 50 wt% S1 slag sintered at 1000 °C, shown in Figure 3b, exhibits iron-rich phases (light spots) surrounded by a light halo. In our opinion, this could be due to iron diffusion in the borosilicate glass, further evidence of glass/slag interactions.

The large pores, visible especially with 50 wt% S1, at 1000 °C (Figure 3b), are hardly explainable as derived from incomplete sintering. As reported above, the firing at 1000 °C could favor some oxygen release, from the reduction of iron oxides; magnetite could not be simply a product of the oxygenolysis of fayalite, but also a product of hematite reduction:

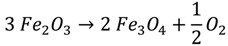
(3)


**Figure 4:**
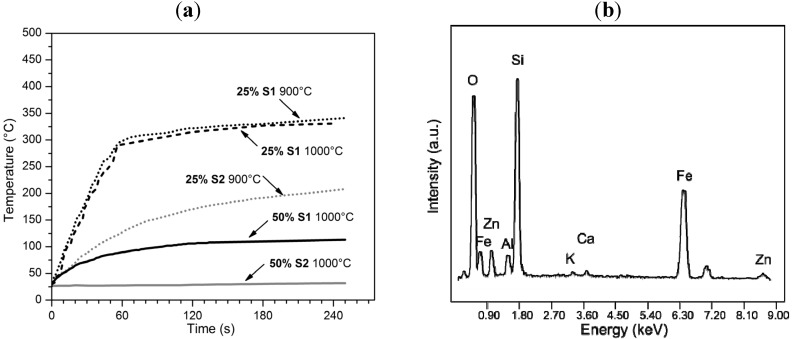
(**a**) Heating of glass ceramics from glass/slag mixtures under alternating magnetic field (AMF); (**b**) EDS spectrum of slag-derived aggregate in the sample with 50 wt% S2, sintered at 1000 °C.

The phase development in the developed glass ceramics suggests a possible application as tiles with a specific functionality. In fact, all of the samples feature magnetite, *i.e.*, a ferrimagnetic phase. As presented in several reports concerning hyperthermia applications of ferrimagnetic materials [30,31], when an alternating magnetic field (AMF) is applied to a ferrimagnetic material, the non-linearity and the delay of its magnetization with respect to the applied magnetic field originate a distinctive hysteresis loop; energy is dissipated, in the form of heat, for every cycle, *i.e.*, for each alternation in the value of external field. Figure 4a illustrates that the developed glass ceramics effectively exhibited, for the samples with low slag content (25 wt% S1 and 25 wt% S2), an intensive heating when subjected to AMF. More precisely, the sample from S1 slag, sintered at 900 °C, reached 300 °C after only 60 s of application. Interestingly, the samples did not exhibit any cracking upon cooling, when AMF was switched off. The resistance to thermal shocks was probably favored by the relatively low thermal expansion of the adopted borosilicate glass (5.5 × 10^−6^ °C^−1^ [18]) and by the porosity (reducing the elastic modulus). In our opinion, these findings could be the basis for the valorization of metallurgical slags, coupled with borosilicate glass, in the manufacturing of innovative heating elements, e.g., parts of cooking tops. Indeed, the fabrication of magnetic glass ceramics from waste has been exploited in the past [4,32,33,34], but no detailed investigation on heat generation under AMF has been presented.

The samples with high slag content presented poor (50 wt% S1) or even no (50 wt% S2) temperature increase, despite the presence of magnetite. This phenomenological “anomaly” could be justified by the observed ionic inter-diffusion, as discussed earlier. Samples treated at 1000 °C did not feature pure magnetite, but a solid solution, comprising ions, such as Zn^2+^, as demonstrated by the EDS spectrum in Figure 4b. Ferrites, *i.e.*, compounds with the general formula M^2+^O∙Fe_2_O_3_, are known to exhibit lower heating rates compared to magnetite [35]. The formation of a Zn-containing solid solution, instead of pure magnetite, could be the reason also for the behavior of the sample containing 25% S1 slag, sintered at 1000 °C; despite the more intense peaks of ferrimagnetic phase (and no evidence of hematite) in Figure 2b, it was similar, in its electromagnetic heating behavior, to the sample from S1 slag, sintered at 900 °C. 

**Figure 5:**
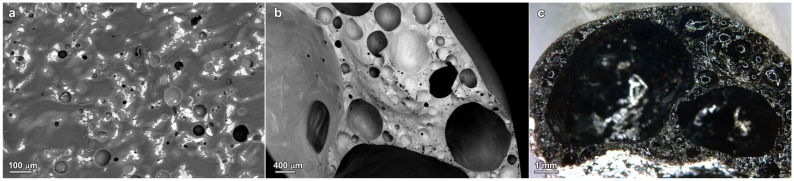
Microstructural details of selected glass ceramics (**a**) 25 wt% S1, 1000 °C; (**b**,**c**) 25 wt% S2, 1000 °C (image (**c**) is from optical stereomicroscopy).

The interdiffusion, at 1000 °C, between glass and slags, even in a low concentration (25 wt%), is testified by Figure 5. The SEM image in Figure 5a, referring to the sample with 25 wt% S1, clearly shows more “isolated” light spots (rich in iron), than in the case of the sample with the same composition sintered at 900 °C (Figure 4a), but also wide diffusion halos around the spots, like in the sample sintered at the same temperature, with higher slag content. The interdiffusion is so extensive for the sample with 25 wt% S2, that halos turn into “streaks”, evident in Figure 5b, around big pores. The big pores, for this sample, as written above, actually correspond to a foaming effect, further evidenced by the optical stereomicroscope image in Figure 5c.

### 3.3. Chemical Stability and Cytotoxicity Studies

The most interesting samples, *i.e.*, those exhibiting intensive heating, for each type of slag (25 wt% S1 and 25 wt% S2), were evaluated also in terms of chemical stability, a property essential for high value applications. The TCLP test was applied as a preliminary approach. As reported in Table 3, the contents of toxic elements were all well below the limits for inert materials. Although successful, the TCLP test was not considered as a definitive proof of inertness, since it is intended mainly for the certification of wastes, rather than for the characterization of products. In addition, antimony (Sb) was not determined, owing to problems of instrument calibration.

**Table 3:** Chemical analysis of the leachate of samples subjected to Toxicity Control Leaching Procedure (TCLP) testing (EN, European standard; * data not determined; **^#^** Liquid/Solid ratio=10 liters/Kg).

Element	Leachate (ppm)		
Sample	25 wt% S1 900 °C	25 wt% S2 900 °C	EN Limits ^#^
As	<0.0049	<0.0049	0.5
Ba	0.0029	0.0538	20
Cd	<0.0002	<0.0002	0.04
Cr	<0.0004	<0.0004	0.5
Cu	<0.0001	<0.0001	2
Hg	<0.0004	<0.0004	0.01
Mo	<0.0033	0.0048	0.5
Ni	0.0302	0.0166	0.4
Pb	<0.0047	0.0077	0.5
Sb *	n.d.	n.d.	0.06
Se	<0.0122	<0.0122	0.1
Zn	<0.0203	<0.0203	4

The assessment of the biocompatibility of waste-derived products by cell biology investigations, although being of high relevance to certify the safety of these materials for general use [36], has been considered only to a limited extent in the past [37]. As discussed recently [38], there is therefore increasing interest to provide data about the safety of waste-derive products based on established cell-culture-based cytotoxicity studies. In this investigation, MEF cell cultures were considered to analyze the possible cytotoxic effects of the glass ceramics produced. Generally, MEF cells are applied in the characterization of biomaterials, e.g., materials for use in medical applications. In this study, the iron-containing glass ceramics were subjected to cell culture tests, and the results were compared with those on materials considered to be safe for every-day use, *i.e.*, soda-lime glass.

Mitochondrial activity was considered as an index of the toxicity level of the examined materials. Soda-lime glass was taken as a reference as a well-known “safe” product. Its mean mitochondrial activity was set at 100% ± standard deviation.

**Figure 6:**
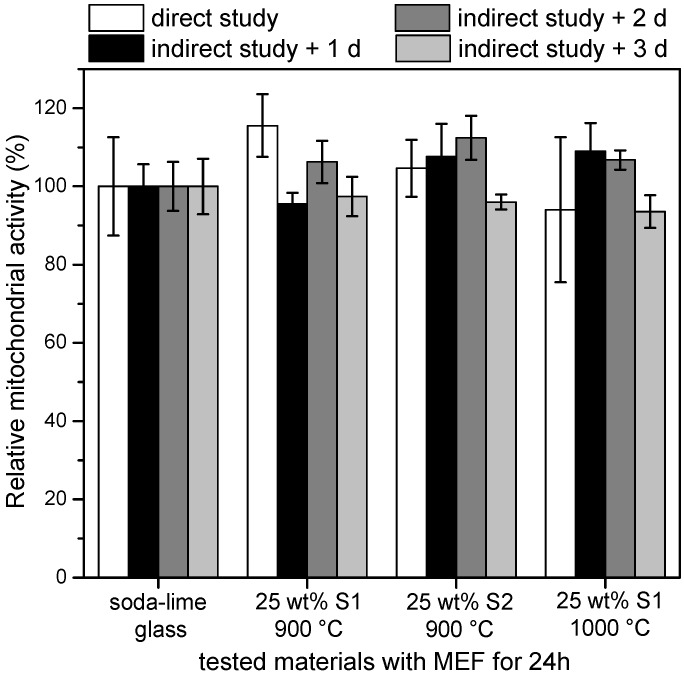
Relative cell viability of slag/glass samples, compared with soda-lime. MEF, mouse embryonic fibroblast.

Figure 6 illustrates the relative mitochondrial activity of MEF cells after 24 h of seeding on waste-derived glass ceramics and soda-lime glass, according to both direct and indirect studies. It can be noted that the mitochondrial activity of samples was practically equivalent to that of the reference glass. During the direct study, sample topology directly impacted on cell adherence. The higher values found for samples 25 wt% S1 900 (113% ± 9%) and 25 wt% S2 900 (106% ± 8%) could be attributed to porosity, which could favor the adhesion of fibroblasts. During the indirect study (black and grey bars), cells seeded on microplates were in contact with ions in the medium, released by samples after 1, 2 and 3 days of dilution. The fluctuation of values was not interpreted further, but it might depend on parameters, such as diffusion rate and concentrations of ions in the medium, synergies and interfacial cell/ion biological reactions. Only a significant drop of mitochondrial activity (not observed) could be reasonably attributed to environmental toxicity. In fact, the non-toxicity value limit value could be set at 50% mitochondrial activity, relative to the reference glass, according to previous toxicology studies [39]. We can therefore state that, under the conditions of the applied test, none of the samples presented toxicity risk, even after three days (indirect study), which was accepted as a significant period to indicate cytotoxicity, considering the quasi-instantaneous reactivity of MEFs to their environment [18,39]. Furthermore, no significant change is observed with increasing firing temperature (the similarity of samples with 25 wt% S1, sintered at 900 or 1000 °C, besides in electromagnetic heating, is confirmed in terms of chemical stability).

**Figure 7:**
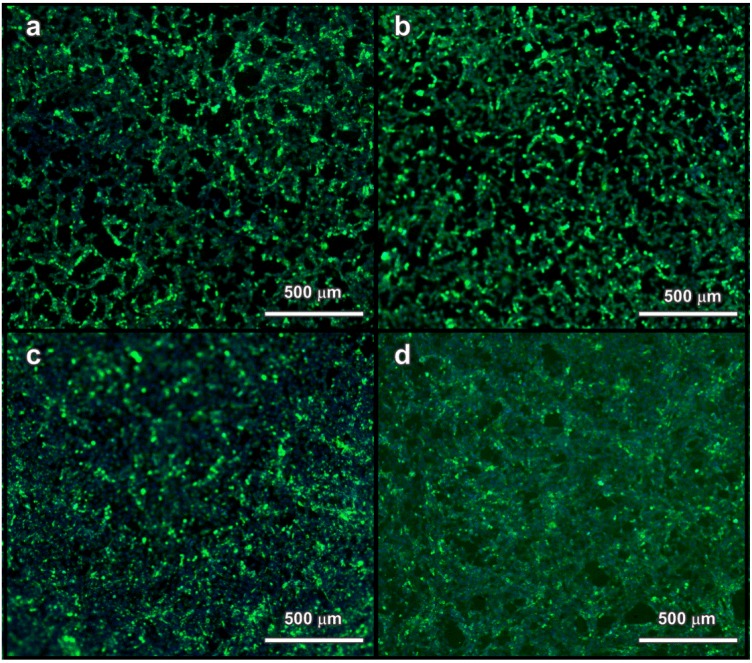
Fluorescence microscope images of samples from direct test method (**a**) 25 wt% S1 900 °C; (**b**) 25 wt% S2 900 °C; (**c**) 25 wt% S1 1000 °C; (**d**) soda-lime glass (reference) (with superposition nucleus (blue) and cell body (green)).

As illustrated by Figure 7, glass ceramic samples, as well as soda-lime glass were fully covered by MEF cells. The cells exhibited spreading and mutual interconnections, with an elongated morphology, which are well-recognized features to indicate biocompatibility [18,36,39]. The glass ceramics, as shown in Table 1, actually contained several elements promoting cellular activity, such as Si, O, Na, K and Ca. In addition, glasses doped with Zn, B, Mg or Fe have shown stimulating effects on cell growth; other minor elements (Al, Cr) are effectively toxic [19]. The results of MEF activity indicate that the diffusion rate of toxic elements in the cell culture medium was reasonably limited. In other words, the chemical stability of the adopted borosilicate glass, employed in the pharmaceutical industry, was not degraded by the incorporation of slags in the present glass ceramics.

## 4. Conclusions

The results obtained and discussed in this paper lead to the following conclusions:

- Metallurgical slags were successfully sintered, mixed with recycled borosilicate glass, at temperatures not exceeding 1000 °C. The developed glass ceramics, owing to the negligible water absorption, could be used as low-cost lightweight tiles;
- Fe-rich phases developed according to slag/glass interactions;
- Owing to the presence of magnetite, the developed glass ceramics (for a slag concentration of 25 wt%), exhibit intensive heating when subjected to an alternating magnetic field, so that they could be applied as novel heating elements;
- The chemical durability of the glass ceramics was assessed by TCLP testing, while the materials biocompatibility was confirmed by cytotoxicity tests.

## References

[1] Lee W.E. (2006). The contribution of ceramics to environmental clean up. Adv. Appl. Ceram..

[2] Rawlings R.D., Wu J.P., Boccaccini A.R. (2006). Glass ceramics: Their production from wastes—A Review. J. Mat. Sci..

[3] Höland W., Beall G.H. (2002). Glass Ceramic Technology.

[4] Colombo P., Brusatin G., Bernardo E., Scarinci G. (2003). Inertization and reuse of waste materials by vitrification and fabrication of glass based products. Curr. Opin. Solid State Mater. Sci..

[5] Francis A.A., Rawlings R.D., Sweeney R., Boccaccini A.R. (2002). Processing of coal ash into glass ceramic products by powder technology and sintering. Glass Technol..

[6] Dimech C., Cheeseman C.R., Cook S., Simon J., Boccaccini A.R. (2008). Production of sintered materials from air pollution control residues from waste incineration. J. Mater. Sci..

[7] Bernardo E., Dal Maschio R. (2001). Glass ceramics from vitrified sewage sludge pyrolysis residues and recycled glasses. Waste Manag..

[8] Chinnam R.K., Francis A.A., Will J., Bernardo E., Boccaccini A.R. (2013). Review. Functional glasses and glass ceramics derived from iron rich waste and combination of industrial residues. J. Non-Cryst. Sol..

[9] Bretcanu O., Spriano S., Verné E., Coïsson M., Tibero P., Allia P. (2005). The influence of crystallised Fe_3_O_4_ on the magnetic properties of coprecipitation-derived ferrimagnetic glass ceramics. Acta Biomater..

[10] Bretcanu O., Verné E., Coïsson M., Tibero P., Allia P. (2006). Magnetic properties of the ferrimagnetic glass ceramics for hyperthermia. J. Magn. Magn. Mater..

[11] Deatsch A.E., Evans B.A. (2014). Heating efficiency in magnetic nanoparticle hyperthermia. J. Magn. Magn. Mater..

[12] Gorai B., Jana R.K., Premchand. (2003). Characteristics and utilisation of copper slag—A review. Res. Conserv. Recycl..

[13] Mihailova I., Mehandjiev D. (2010). Characterization of fayalite from copper slags. J. Univ. Chem. Technol. Metal..

[14] Alp I., Deveci H., Süngün H. (2008). Utilization of flotation wastes of copper slag as raw material in cement production. J. Hazard. Mater..

[15] Francis A.A. (2007). Magnetic characteristics of iron-containing glass originated from the mixture of various wastes. Ceram. Int..

[16] Coruh S., Ergun O.N., Cheng T.-W. (2006). Treatment of copper industry waste and production of sintered glass ceramic. Waste Manag. Res..

[17] Zhihong Y., Qiao L., Jixiang X., Yong H., Guangdong L., Yi K. (2004). Preparation and crystallization of glass-ceramics derived from iron-rich copper slag. J. Alloys Comp..

[18] Bernardo E., Scarinci G. (2004). Sintering behaviour and mechanical properties of Al_2_O_3_ platelet-reinforced glass matrix composites obtained by powder technology. Ceram. Int..

[19] Hoppe A., Güldal N.S., Boccaccini A.R. (2011). A review of the biological response to ionic dissolution products from bioactive glasses and glass ceramics. Biomaterials.

[20] Bernardo E. (2008). Fast sinter-crystallization of a glass from waste materials. J. Non-Cryst. Sol..

[21] Bernardo E., Bonomo E., Dattoli A. (2010). Optimisation of sintered glass–ceramics from an industrial waste glass. Ceram. Int..

[22] Ettler V., Johan Z., Touray J.C., Jelínek E. (2000). Zinc partitioning between glass and silicate phases in historical and modern lead-zinc metallurgical slags from the Příbram district, Czech Republic. C. R. de l’Acad. des Sci.-Ser. IIA.

[23] Raghavan V. (2010). Phase Diagram Evaluations—Fe-O-Si-Zn (Iron-Oxygen-Silicon-Zinc). J. Phase Equilibria Diffus..

[24] O’Neill H.St.C. (1987). Quartz-fayalite-iron and quartz-fayalite-magnetite equilibria and the free energy of formation of fayalite (Fe_2_SiO_4_) and magnetite (Fe_3_O_4_). Am. Mineral..

[25] Appendino P., Ferraris M., Matekovits I., Salvo M. (2004). Production of glass-ceramic bodies from the bottom ashes of municipal solid waste incinerators. J. Eur. Ceram. Soc..

[26] Shelby J.E. (2005). Introduction to Glass Science and Technology.

[27] Thomas E.S., Thompson J.G., Withers R.L., Sterns M., Xiao Y., Kirkpatrick R.J. (1994). Further Investigation of the Stabilization of β-Cristobalite. J. Am. Ceram. Soc..

[28] Cheng T.W., Huang M.Z., Tzeng C.C., Cheng K.B., Ueng T.H. (2007). Production of coloured glass ceramics from incinerator ash using thermal plasma technology. Chemosphere.

[29] Cheng T.W., Chen Y.S. (2004). Characterisation of glass ceramics made from incinerator fly ash. Ceram. Int..

[30] Andreu I., Natividad E. (2013). Accuracy of available methods for quantifying the heat power generation of nanoparticles for magnetic hyperthermia. Int. J. Hyperth..

[31] Jordan I., Wust P., Fahling H., John W., Hinz A., Felix R. (2009). Inductive heating of ferrimagnetic particles and magnetic fluids: Physical evaluation of their potential for hyperthermia. Int. J. Hyperth..

[32] Francis A.A. (2006). Crystallization kinetics of magnetic glass–ceramics prepared by the processing of waste materials. Mater. Res. Bull..

[33] Francis A.A., Rawlings R.D., Sweeney R., Boccaccini A.R. (2004). Crystallization kinetic of glass particles prepared from a mixture of coal ash and soda-lime cullet glass. J. Non-Cryst. Sol..

[34] Rosenweig R.E. (2002). Heating magnetic fluid with alternating magnetic field. J. Magn. Magn. Mater..

[35] Sandu V., Nicolescu M.S., Kuncser V., Damian R., Sandu E. (2012). Magnetic glass ceramics. J. Adv. Ceram..

[36] Pollard T.D., Earnshaw W.C., Lippincott-Schwartz J. (2008). Cell Biology.

[37] Boccaccini A.R., Petitmermet M., Wintermantel E. (1997). Glass ceramics from Municipal Incinerator Fly Ash. Ceram. Bull..

[38] Ponsot I., Bernardo E., Bontempi E., Depero L., Chinnam R.K., Detsch R., Boccaccini A.R. (2014). Recycle of pre-stabilized municipal waste incinerator fly ash and soda-lime glass into sintered glass ceramics. J. Clean. Prod..

[39] Trevan J.W., JSTOR (1927). The Error of Determination of Toxicity. Series B, Containing Papers of a Biological Character, Proceedings of the Royal Society of London, 1927.

